# Want to Engage More Physical Therapy Students in Geriatrics? Teach the 4Ms

**DOI:** 10.1093/geroni/igaa057.048

**Published:** 2020-12-16

**Authors:** Tiffany Shubert, Cristine Henage

**Affiliations:** 1 Shubert Consulting, Chapel Hill, North Carolina, United States; 2 UNC School of Medicine, Chapel Hill, North Carolina, United States

## Abstract

In 2019, 1.2% of the 209,000 licensed physical therapists in the United States had completed sufficient training to be Geriatric Certified Specialists. The dramatic demographic shift in the population will require all physical therapists to have foundational knowledge of evidence-based management of older adults. Our purpose was to pilot the impact of an 8-week curriculum for physical therapy students that integrated key concepts of rehabilitation for older adults with the Age-Friendly Health System’s 4Ms (Mentation, Mobility, Medications, What Matters). The curriculum included guest speakers from medicine, social work, nutrition, pharmacy, community providers (YMCA) and older adults. Every class modeled how to assess the Ms and integrate information into a plan aligned with what matters to the client. Students completed a pre-post survey to evaluate their understanding of the 4Ms, and self-assess confidence in applying concepts to practice. Results supported the value of integrating the 4Ms into the curriculum. Over 89% of respondents reported assessing medications and mentation was very important to patient care compared to 11% and 33% pre-course, and 78% of students reporting they were highly-likely to advocate for the 4Ms as part of their practice. The 4Ms provided a framework that made geriatric care more appealing. Several students commented they had no interest in geriatrics prior to the course, but were more confident in their abilities and more interested in caring for older adults. Findings from this pilot support the value of the 4Ms as a framework for graduate-level allied health programs curriculum development.

